# Molecular Characterization of Peroxisome Proliferator-Activated Receptor-Gamma Coactivator-1α (PGC1α) and Its Role in Mitochondrial Biogenesis in Blunt Snout Bream (*Megalobrama amblycephala*)

**DOI:** 10.3389/fphys.2018.01957

**Published:** 2019-01-24

**Authors:** Xiaojun Song, Samad Rahimnejad, Wenhao Zhou, Linsen Cai, Kangle Lu

**Affiliations:** ^1^Laboratory for Animal Nutrition and Immune Molecular Biology, College of Life Sciences, Qingdao Agricultural University, Qingdao, China; ^2^Laboratory of Aquatic Animal Nutrition and Physiology, Fisheries College, Jimei University, Xiamen, China; ^3^South Bohemian Research Center of Aquaculture and Biodiversity of Hydrocenoses, Institute of Aquaculture and Protection of Waters, Faculty of Fisheries and Protection of Waters, University of South Bohemia in České Budějovice, České Budějovice, Czechia

**Keywords:** blunt snout bream, *PGC1*α, molecular characterization, mitochondrial biogenesis, gene cloning

## Abstract

*PGC1*α is a transcriptional coactivator that plays key roles in mitochondrial biogenesis, so exploring its molecular characterization contributes to the understanding of mitochondrial function in cultured fish. In the present study, a full-length cDNA coding *PGC1*α was cloned from the liver of blunt snout bream (*Megalobrama amblycephala*) which covered 3741 bp with an open reading frame of 2646 bp encoding 881 amino acids. Sequence alignment and phylogenetic analysis revealed high conservation with other fish species, as well as other higher vertebrates. Comparison of the derived amino acid sequences indicates that, as with other fish, there is a proline at position 176 (RIRP) compared to a Thr in the mammalian sequences (RIRT). To investigate PGC1α function, three *in vitro* tests were carried out using primary hepatocytes of blunt snout bream. The effect of AMPK activity on the expression of *PGC1*α was determined by the culture of the hepatocytes with an activator (Metformin) or inhibitor (Compound C) of AMPK. Neither AMPK activation nor inhibition altered *PGC1*α expression. Knockdown of *PGC1*α expression in hepatocytes using small interfering RNA (si-RNA) was used to determine the role of PGC1α in mitochondrial biogenesis. No significant differences in the expression of *NRF1* and *TFAM*, and mtDNA copy number were found between control and si-RNA groups. Also, hepatocytes were cultured with oleic acid, and the findings showed the significant reduction of mtDNA copy number in oleic acid group compared to control. Moreover, oleic acid down-regulated the expression of *NRF1* and *TFAM* genes, while *PGC1*α expression remained unchanged. Our findings support the proposal that *PGC1*α may not play a role in mitochondrial biogenesis in blunt snout bream hepatocytes.

## Introduction

The peroxisome proliferator-activated receptor-gamma coactivator 1 (PGC1) family members are transcriptional coactivators that play key roles in the transcriptional control of mitochondrial biogenesis and respiratory function (Scarpulla, 2011). PGC1α was the first characterized PGC1 family member being identified as a stimulator of thermogenin expression in brown adipose tissue of mice (Puigserver et al., 1998). The other family members including PGC1β and PGC1-related coactivator (PRC) were subsequently discovered through database searches for PGC1α homologs. These three PGC1 proteins share some features but they also have distinct functions. PGC1α and PGC1β share several activation roles within the cell including developmental differentiation in mitochondrial biogenesis and adaptation to external stimuli such as nutrition and exercise (Goto et al., 2000; Mortensen et al., 2006; Uldry et al., 2006).

PGC1α has the capability to bind different factors in an independent manner, which enables it to act as a regulator for mitochondrial biogenesis in mammals and birds (Puigserver and Spiegelman, 2003; Handschin and Spiegelman, 2006; Vercauteren et al., 2008). Although the role of PGC1α in mitochondrial biogenesis of mammals and birds has been well established, it still remains to be elucidated in fish (Bremer et al., 2016). In mammalian cells, the primary upstream kinase of PGC1α is AMP-activated protein kinase (AMPK), which has also been identified as a regulator of mitochondrial biogenesis. Further, activated PGC1α in mammals often stimulates the transcription of numerous genes associated with mitochondrial biogenesis such as nuclear respiratory factor 1 (*NRF1*) and mitochondrial transcription factor A (*TFAM*). The AMPK-PGC1α-NRF1 pathway is well established in mammalian models, but this axis remains unclear in fish. Interestingly, the findings of a recent study indicated that the AMPK-PGC1α-NRF1 may be disrupted in fish (Bremer et al., 2016). This may indicate that different species probably adopt distinct solutions for controlling mitochondrial biogenesis. From an evolutionary point of view, duplication events and mutations often induce divergence/diversity in function of genes between fish and mammals (Sémon and Wolfe, 2007).

In cultured fish, intake of excessive fat often impairs mitochondrial biogenesis and causes dysfunction of lipid metabolism (Lu et al., 2013a,b). Blunt snout bream (*Megalobrama amblycephala*) is an herbivorous freshwater fish native to China. It has been a favorable fish for aquaculture in China because of its fast growth, tender flesh, and high disease resistance. However, incidence of mitochondrial dysfunction has been a common phenomenon in its farming in captivity which is associated with oxidative stress and apoptosis (Lu et al., 2014). The objectives of this research were to (1) achieve the molecular characterization of PGC1α, (2) elucidate AMPK-PGC1α-NRF1 axis in mitochondrial biogenesis, and (3) explore the role of *PGC1*α in controlling mitochondria content.

## Materials and Methods

### Experimental Fish and Sample Collection

All the fish were treated in accordance with the principles of the Basel Declaration and Recommendations of Animal Research Institute Committee guidelines, Jimei University, China. Blunt snout bream juveniles were obtained from a private hatchery (Guangzhou, China), transported to the Fisheries laboratory of Jimei University, and cultured in a recirculating aquaculture system under the following conditions: water temperature, 25–27°C; DO, 5.0–6.0 mg l^−1^; pH 7.2–7.6. Fish were fed a commercial diet (31% protein, 7% lipid) (ALPHA feed Co. Ltd., China) for 1 week to acclimate them to the experimental conditions. At the end of the acclimation period, fish were used for gene cloning and hepatocyte culture studies. Prior to sampling, fish (weight: 20 ± 1 g) were starved for 24 h and then euthanized with 100 mg l^−1^ of MS-222 (tricaine methanesulfonate; Sigma, United States). Tissue samples including heart, liver, white muscle, red muscle, gills, and adipose tissue were immediately collected, flash frozen in liquid nitrogen and then stored at −80°C.

### *PGC1*α cDNA Cloning

Total RNA was extracted from liver using RNAiso Plus (Takara Co. Ltd., Japan). RNA samples were treated by RQ1 RNase-Free DNase prior to RT-PCR (Takara Co. Ltd., Japan) to avoid genomic DNA amplification. Quantity and purity of isolated RNA were subsequently determined by absorbance measures at 260 and 280 nm, and its integrity was tested by electrophoresis in 1.0% formaldehyde denaturing agarose gels. The partial fragment of *PGC1*α was obtained by high throughput sequencing as described earlier (Zhang et al., 2015), and added to the National Institutes of Health’s Short Read Archive database (accession no. SRX679226). According to the sequence information of this fragment, gene-specific primers were designed for RACE (Supplementary Table S1). Rapid amplification of the 5′ end was performed using the 5′ RACE System of Invitrogen. Briefly, 2 μg sample RNA was used with PGC1α5-R to obtain the first strand cDNA. After RNase treatment, an Oligo (dC) at the 5′ end was added using terminal deoxynucleotidyl transferase. The resulting product was used as a template for the first PCR amplification at 94°C for 2 min and 30 cycles of amplification at 94°C for 30 s, 55°C for 30 s, 72°C for 60 s, and 72°C for 7 min. Then, the first PCR product was used as a template for the nested PCR. The nested PCR product was eluted from 1% agarose gel and delivered to Shanghai Sangon Biotech Service Co. Ltd. (Shanghai, China) for sequencing. Rapid amplification of the 3′ end was performed using the 3′-full RACE Core Set (TaKaRa, Dalian, China) following the manufacturer’s instructions. After the first and nested PCRs (consisted of 25 cycles of 30 s at 94°C, 30 s at 65°C and 1 min at 68°C), the PCR product was eluted from 1.0% agarose gel using a PCR purification kit (QIAGEN, United States) and sequenced.

### Alignment, Phylogenetic and Codon-Based Sequence Analysis

A multiple sequence alignment was performed for homology sequences of PGC1 family members using MUSCLE with default parameters, which were manually curated if necessary. Gene structure and position of motifs were checked by hand using data from Entrez genes, and domain conservation was predicted by SMART software^1^. Phylogenetic analyses were conducted using MrBayes 3.2 for a Bayesian analysis employing a mixed amino acid substitution model. The CODEML program in the PAML4.4 software package was used to analyze changes in selective pressure which allow for variable selection patterns among amino acid sites, M0, M1a (nearly neutral), M2a (positive selection), M7 (beta), M8 (beta and ω), to test for the presence of sites under positive selection. The presence of codons evolving under positive selection was further tested by contrasting the M1a and M2a models, and the M7 and M8 models by likelihood ratio tests (LRTs). To explore the divergence of different branches of *PGC1* family genes in the evolutionary history, a branch model of the CODEML software was used to compute the non-synonymous/synonymous substitution rate ratio of different branches. Finally, positive selection sites in fish *PGC1*α sequences were detected by applying a branch-site model and statistical analysis by Bayes empirical Bayes (BEB) methods.

### Hepatocytes Culture, Treatment and siRNA Transfection

#### Isolation of Hepatocytes

Prior to isolation of hepatocytes, fish were anesthetized with 100 mg l^−1^ of MS-222 and bled by cutting the gill arches. Then, liver was rapidly isolated and washed several times in ice-cold phosphate buffered saline (PBS) containing antibiotic (100 IU ml^−1^ penicillin G sodium and 100 IU ml^−1^ streptomycin). After removal of PBS using sterile pipette, the samples were cut into small pieces (about 1 mm^3^) and digested with pancreatin at 28°C for 30 min. Thereafter cell suspension was centrifuged at 500 *g* for 10 min and washed twice. The harvested cell pellets were re-suspended in Leibovitz’s L-15 medium (L15 medium) (HyClone^TM^, United States) with 15% fetal bovine serum (Biological Industries, United States) at a density of 1 × 10^6^ ml^−1^. For each test three different fish were used and each time the livers were pooled to make a single sample. The viability of hepatocytes was assayed before the beginning of each trial using a Cell Counting Kit-8 (CCK-8, Dojindo Laboratories, Kumamoto, Japan).

#### Culturing Hepatocytes With AMPK Activator/Inhibitor

Cells attached and cultured in 2 ml of the following media: control medium (L15), AMPK activated metformin (L15+ 200 μM metformin) and AMPK inhibited Compound C (L15+ 100 μM Compound C). After culturing for 48 h, cells were harvested by trypsinization (0.25% trypsin–EDTA) at 25°C in 5 min for analyzing the expression of *PGC1*α. All the tests were performed in three replicates.

Western blots were used to measure the level of phospho-AMPKα. Briefly, cell pellets (about 10^8^ cells) were lysed in ice-cold lysis buffer (Cell Signaling, Danvers, MA, United States) and centrifuged at 12000 *g* for 5 min, and then the resulting supernatants were stored at −80°C. Total protein was determined according to the methods outlined by Bradford (1976). Aliquots of each sample were added to an equal volume of SDS sample buffer (Laemmli, 1970), boiled for 5 min, and 20 μg of total protein was loaded into each well, separated by SDS-PAGE for 1–2 h at 100 V using a Mini-Protean system (Bio-Rad, Spain) and transferred to a polyvinylidene fluoride (PVDF) membrane (Millipore, Burlington, MA, United States). Subsequently, the membrane was blocked with blocking buffer (20 mM Tris-HCl, 150 mM NaCl, 0.05% Tween-20, pH 7.6) containing 5% (w/v) non-fat dry milk for 1 h. The membrane was then incubated with rabbit polyclonal antibodies against GAPDH blots (Cell Signaling Technology, United States) and antiphospho-AMPKα (#2535, Cell Signaling Technology, United States) at 4°C overnight. After washing, membranes were incubated with anti-rabbit secondary antibody. Bands were visualized by an electro-chemiluminescence (ECL) system (GE Healthcare, Buckinghamshire, United Kingdom) and quantified by the densitometry band analysis tool in ImageJ.

#### Transfection

Hepatocytes were transfected with small interfering RNA (siRNA) duplexes (5′-Chol, 2′-Ome) for PGC1α (si-PGC1α) or negative control (GenePharma), which were named siRNA-PGC1α group and siRNA-NC group, respectively. The sequences of si-PGC1α duplexes were as follows: sense sequence, 5′-GGAUGUCAGUGACCUCGAUTT-3′; anti-sense sequence, 5′-AUCGAGGUCACUGACAUCCTT-3′. The sequences of NC siRNA duplexes were as follows: sense sequence, 5′-UUCUCCGAACGUGUCACGUTT-3′; anti-sense sequence, 5′-ACGUGACACGUUCGGAGAATT-3′. The delivery of siRNA duplexes was carried out using Lipofectamine^®^ RNAiMAX Transfection Reagent (Invitrogen) according to the manufacturer’s instructions. Cells were incubated with siRNA-lipid complex for 48 h, and then harvested to measure the expression of *PGC1*α, *NRF1*, and *TFAM* genes, and mitochondrial content. All the tests were performed in three replicates.

#### Culturing Hepatocytes With Oleic Acid

Two milliliters of isolated hepatocytes was seeded in each well of 6-well culture plates. After 24 h, all cells attached and cultured in 2 ml of the following media: control medium (L15) and oleic acid medium (L15+ 600 μM oleic acid). Oleic acid was purchased from Sigma Chemicals (O1250). After 72 h, the cells were collected for analysis. Then, cells were harvested by trypsinization (0.25% trypsin–EDTA) at 25°C in 5 min to measure the expression of *PGC1*α, *NRF1* and *TFAM* genes, and mitochondrial content. All the tests were performed in three replicates.

### Gene Expression

Quantitative real-time PCR (qPCR) method was used to determine the mRNA abundance as gene expression. Expression of *PGC1*α in six tissues including liver, white muscle, red muscle, heart, fat tissue, and gill was measured *in vivo* test. Expression of *PGC1*α, *NRF1* and *TFAM* in cultured hepatocytes was also determined by qPCR.

Extraction of total RNA and first strand cDNA synthesis were performed as described above. Real-time PCR was employed to determine mRNA abundance based on the SYBR Green I fluorescence kit. Primer characteristics used for real-time PCR are listed in Supplementary Table S1, according to the MIQE Guidelines (Bustin et al., 2011). Real-time PCR was performed in a Mini Option real-time detector (Bio-Rad, United States). The fluorescent quantitative PCR reaction solution consisted of 12.5 μl SYBR^®^ premix Ex TaqTM (2×), 0.5 μl PCR forward primer (10 μM), 0.5 μL PCR reverse primer (10 μM), 2.0 μL RT reaction (cDNA solution), and 9.5 μL ddH_2_O. The reaction conditions were as follows: 95°C for 3 min followed by 45 cycles consisting of 95°C for 10 s and 60°C for 20 s. The fluorescent flux was then recorded, and the reaction continued at 72°C for 3 min. The dissociation rate was measured between 65 and 90°C. Each increase of 0.2°C was maintained for 1 s, and the fluorescent flux was recorded. All amplicons were initially separated by agarose gel electrophoresis to ensure that they were of correct size. A dissociation curve was determined during the PCR program to make sure that specific products were obtained in each run. At the end of the reaction, the fluorescent data were converted into Ct values. Each expression level was normalized to ribosomal protein L13a (Rpl13a) using the 2^−ΔΔCT^ method without correction for primer efficiency.

### Mitochondrial Content Assay

Mitochondrial content is typically measured directly through qPCR quantitation of mitochondrial DNA (mtDNA) copies expressed relative to nDNA (Reverter et al., 2017). DNA was extracted using Qiagen DNeasy tissue ‘on column’ system as per manufacturer’s instructions. The mtDNA content was expressed relative to nDNA copy number (Reverter et al., 2017). Primers for mtDNA (ND1) were: forward primer 5′ TAGCCCCTGCCTGACCACT 3′, reverse primer 5′ CTGGGATGTGGTGAATGTGTGA 3′, and for nDNA (beta-globin): forward primer 5′ GAATGCTCATCGTCTACCCTCA 3′, reverse primer 5′ ATGGCTGTCATCACAGTTTTGC 3′. The mtDNA copy number was calculated using real time PCR method as described earlier (Sun et al., 2012).

### Statistical Analysis

Data were analyzed by SPSS 16.0 for Windows software (SPSS, Chicago, IL, United States). One-way analysis of variance (ANOVA) with *post hoc* multiple comparison by Student–Newman–Keuls test was used to analyze differences of data from three groups. Student’s *t*-test was used to analyze differences of data from two groups. The level of significance was set at *P* < 0.05. All data were presented as means ± standard error of the mean (SEM).

## Results

### Characterization of PGC1α

**FIGURE 1:**
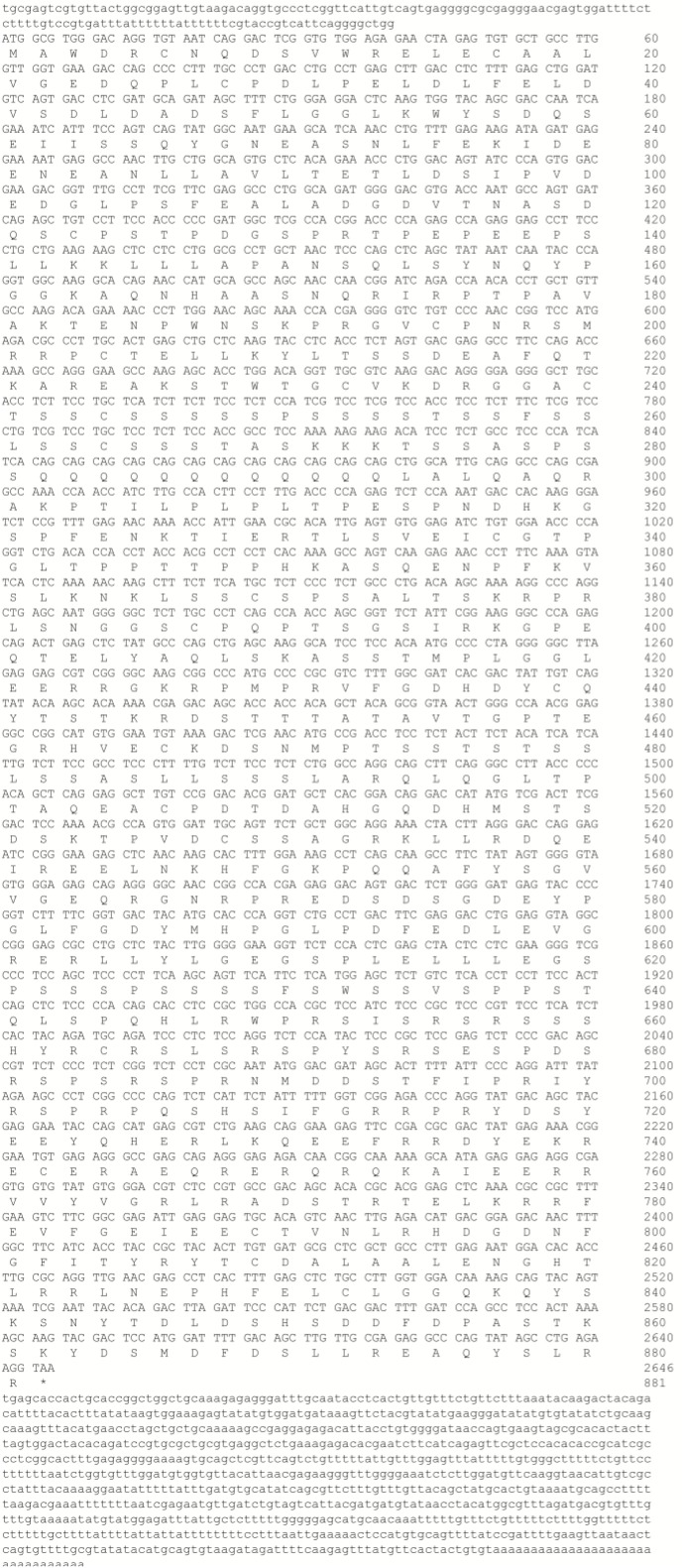
The nucleotide sequence of the *PGC1*α cDNA in *M. amblycephala*, and the deduced amino acid sequence. Uppercase letters indicate the translated region, whereas lowercase letters represent the un-translated region.

**FIGURE 2:**
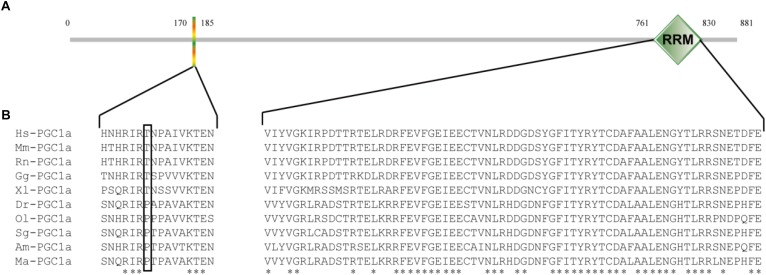
The sequence analysis of *M. amblycephala* PGC1α. (A) The domain structure of *M. amblycephala* PGC1α protein by SMART software, which contains a RRM domain only. (B) The multiple sequence alignment analysis of PGC1α proteins among *M. amblycephala* and other vertebrate animals. Hs, *Homo sapiens*; Mm, *Mus musculus*; Rn, *Rattus norvegicus*; Gg, *Gallus gallus*; Xl, *Xenopus laevis*; Dr, *Danio rerio*; Ol, *Oryzias latipes*; Sg, *Sinocyclocheilus graham*; Am, *Astyanax mexicanus*; Ma, *M. amblycephala*.

The full-length *PGC1*α cDNA sequence was submitted to Genbank (accession number: MH791034). This full-length cDNA covered 2646 bp of open reading frame (ORF) encoding an 881 AA polypeptide, 138 bp of 5′-untranslated region (UTR) and 957 bp of 3′-UTR, and contained a canonical polyadenylation signal (AATAA) (Figure 1). Furthermore, analysis by SMART software showed that *M. amblycephala* PGC1α protein also contained an RRM domain in C-terminal (Figure 2A). Recent studies showed that PGC1 proteins could be phosphorylated though the conserved sequence (HNHRIRTNPAIVKTE) in N-terminal of human. The results of multiple alignment showed that there was a difference in the position of the Thr in the snout bream sequence (RIRPTP), as well as compared to the mammalian sequence (RIRTNP). Since the Thr is an important AMPK-phosphorylation site within the human sequence, this presence of the proline and the absence of the Thr in the other fish sequences may indicate a difference in the function of PGC1α between fish and humans (Figure 2B).

**FIGURE 3:**
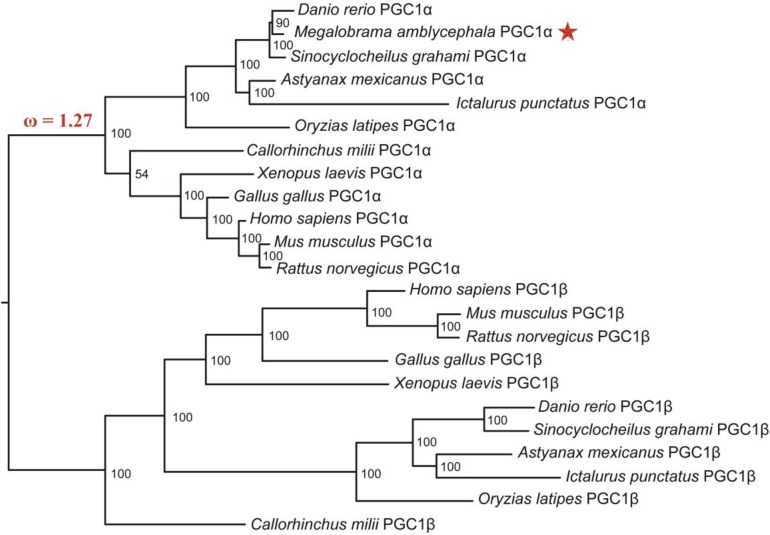
Phylogenetic tree of PGC1 family proteins including *M. amblycephala* PGC1α protein (marked by star on the right of the evolution tree). The Bayesian analysis results showed that the PGC1 family proteins were classified into two groups: PGC1α group and PGC1β group with high bootstrap values. The ω value on the branch of phylogenetic tree represents non-synonymous/synonymous substitution rate (ω = dn/ds), which provides a measurement for the change of selective pressure.

To explore the functional divergence of PGC1α between mammals and *M. amblycephala*, the CODEML program of PAML4.4 software package was used to analyze the changes in selective pressure of PGC1 family proteins in the evolutionary history. Although the PGC1 family members underwent very strong purifying selection (ω = 0.23) (Table 1), a branch underwent positive selection when the fish evolved to amphibians, birds and mammals (ω = 1.27) (Figure 3). Nine positive selection sites were detected by M7 vs. M8 model (Table 1). We used branch-site model to examine whether specific positive selection sites exist in fish, and the results showed that there are 43 positive selection sites.

**Table 1:** Likelihood values and parameter estimates of computing position selection site by site-specific model and branch-site model for the PGC1 family members.

Model	lnL	Parameter	Positive selection site	2ΔL	LRT
M0 (one rate)	−45490.081830	ω = 0.23	None		
M1a (neutral)	−44821.792380	ω0 = 0.15521, *p*0 = 0.60168	Not allowed	13365789	*P* < 0.001
		ω1 = 1.00000, *p*1 = 0.39832		(M1a vs. M2a)	
M2a (selection)	−44821.792380	ω0 = 0.15521, *p*0 = 0.60168	Not found		
		ω1 = 1.00000, *p*1 = 0.34869			
		ω2 = 1.00000, *p*2 = 0.04963			
M7 (beta)	−44410.000671	*p* = 0.81186, *q* = 1.86945	Not allowed		
M8 (beta and ω)	−44403.662859	*p*0 = 0.99546, *p* = 0.81701,	28 D, 443 T, 953 T, 966 E, 968 S,	12.675624	*P* < 0.01
		*q* = 1.90879, *p*1 = 0.00454	973 K, 975 V, 977 P, 1035 I	(M7 vs. M8)	
		ω = 79.91184			
Branch-site model	−44793.494406	Background:			
		ω0 = 0.14696, ω1 = 1.00000	237 S, 272 I, 281 Q, 294 T, 575 Q,		
		ω2a = 0.14696, ω2b = 1.00000	865 S, 872 I, 874 I, 875 N^∗∗^, 878 Q,		
		foreground:	899 L, 973 K, 1030 K^∗^, 1032 P^∗^,		
		ω0 = 0.14696, ω1 = 1.00000,	1035 I, 1038 G, 1056 S, 1141 P,		
		ω2a = 1.00000, ω2b = 1.00000	1148 R, 1168 R, 1188 P, 1306 S		

*^∗^Means that P < 0.05 by LRT test of BEB analysis, ^∗∗^means that P < 0.01 by LRT test of BEB analysis.*

### Phylogenetic Analyses

To explore the origin and evolutionary history of PGC1 family, Blastp or tBlastn were employed to retrieve the distribution of PGC1 family members in different animals. There is no homologous of PGC1 family in genomes of Cyclostomata, Urochordata, Cephalochordate and other invertebrate (data not shown), but more than two RRM domains present. This may indicate domain rearrangement in the evolutionary history of PGC1 family.

The evolutionary relationships of PGC1 family were revealed by the topology of a phylogenetic tree supported by high bootstrap values. As expected, the PGC1 family proteins were categorized into PGC1α and PGC1β groups. Conservation of PGC1α became evident from the high bootstrap values observed between *M. amblycephala* PGC1α and that of other fish species including Cypriniformes species: *Danio rerio* and *Sinocyclocheilus graham*; Siluriformes species: *Ictalurus punctatus*; and Beloniformes species: *Oryzias latipes* (Figure 3). The phylogenetic relationship based on the PGC1 family proteins was consistent with the traditional classification.

### The Role of *PGC1*α in Controlling Mitochondria Content

**FIGURE 4:**
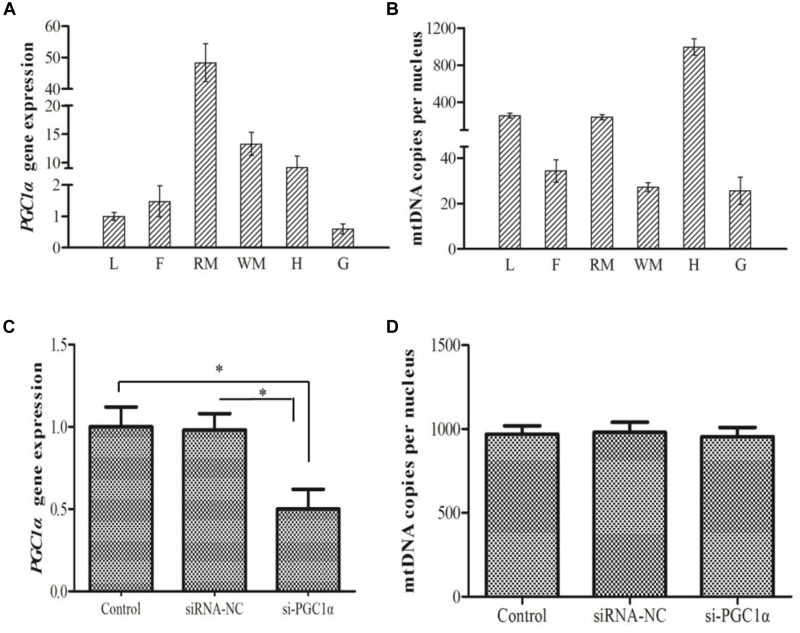
The gene expression of *PGC1*α **(A)** and mtDNA copy number **(B)** in different tissues of *M. amblycephala* (L, liver; F, fat tissue; RM, red muscle; WM, white muscle; H, heart; G, gill). The expression of *PGC1*α **(C)** and mtDNA copy number **(D)** in primary hepatocytes of *M. amblycephala* after the knockdown of *PGC1*α. One-way analysis of variance (ANOVA) with *post hoc* multiple comparison by Student–Newman–Keuls test was used to analyze differences. The asterisk indicate significant difference (*P* < 0.05).

*PGC1*α gene ubiquitously expressed with varying levels in all the tested tissues. As shown in Figure 4A, the highest expression of *PGC1*α was observed in red muscle followed by white muscle, heart, liver, fat tissue, and gill, respectively. The mtDNA copy number of *M. amblycephala* was the highest in heart, moderate in liver and red muscle and low in fat tissue and gill (Figure 4B). A correlation analysis was used to examine whether PGC1α regulates the mitochondrial content in *M. amblycephala*, and the results showed that PGC1α does not regulate the mtDNA copy number in *M. amblycephala* (Pearson’s correlation coefficient: *r* = 0.058, *P* = 0.914; Spearman correlation coefficient: ρ = 0.257, *P* = 0.623).

In order to evaluate the correlation between *PGC1*α expression and mitochondrial content, siRNA technology was used to knockdown expression of *PGC1*α. The expression of *PGC1*α was significantly down-regulated (*P* = 0.001) in si-RNA group compared to control group (Figure 4C). However, there was no significant difference in mtDNA copy number between control and siRNA groups (Figure 4D).

### *PGC1*α Response to AMPK Activation/Inhibition

**FIGURE 5:**
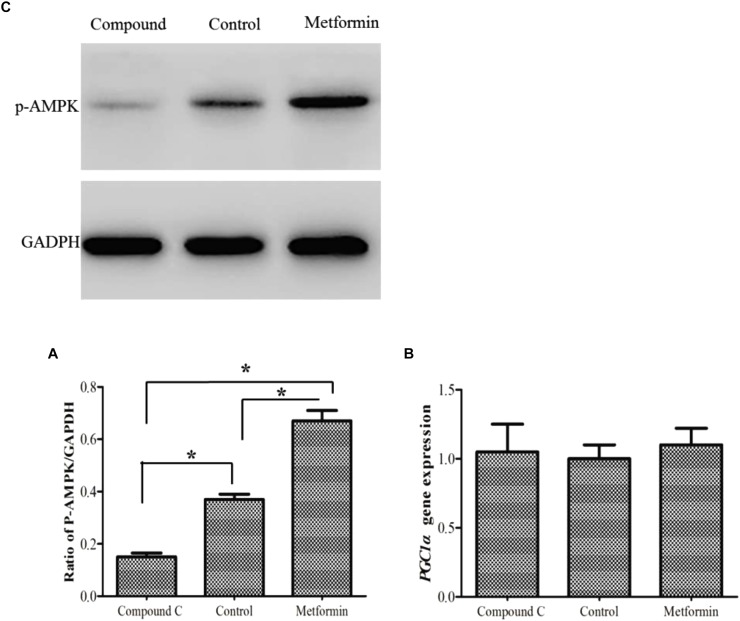
Phospho-AMPK level **(A)** as determined by Western blot in primary hepatocytes of *M. amblycephala* after activation/inhibition. *PGC1*α gene expression **(B)** after AMPK activation/inhibition. One-way analysis of variance (ANOVA) with *post hoc* multiple comparison by Student–Newman–Keuls test was used to analyze differences. The asterisk indicate significant difference (*P* < 0.05).

In this study, metformin/Compound C was used as activator/inhibitor of the AMPK activity. The findings showed that there was no change in *PGC1*α expression following either activation or inhibition of AMPK (Figure 5).

### Expressions of *NRF1* and *TFAM* After Knockdown of *PGC1*α

**FIGURE 6:**
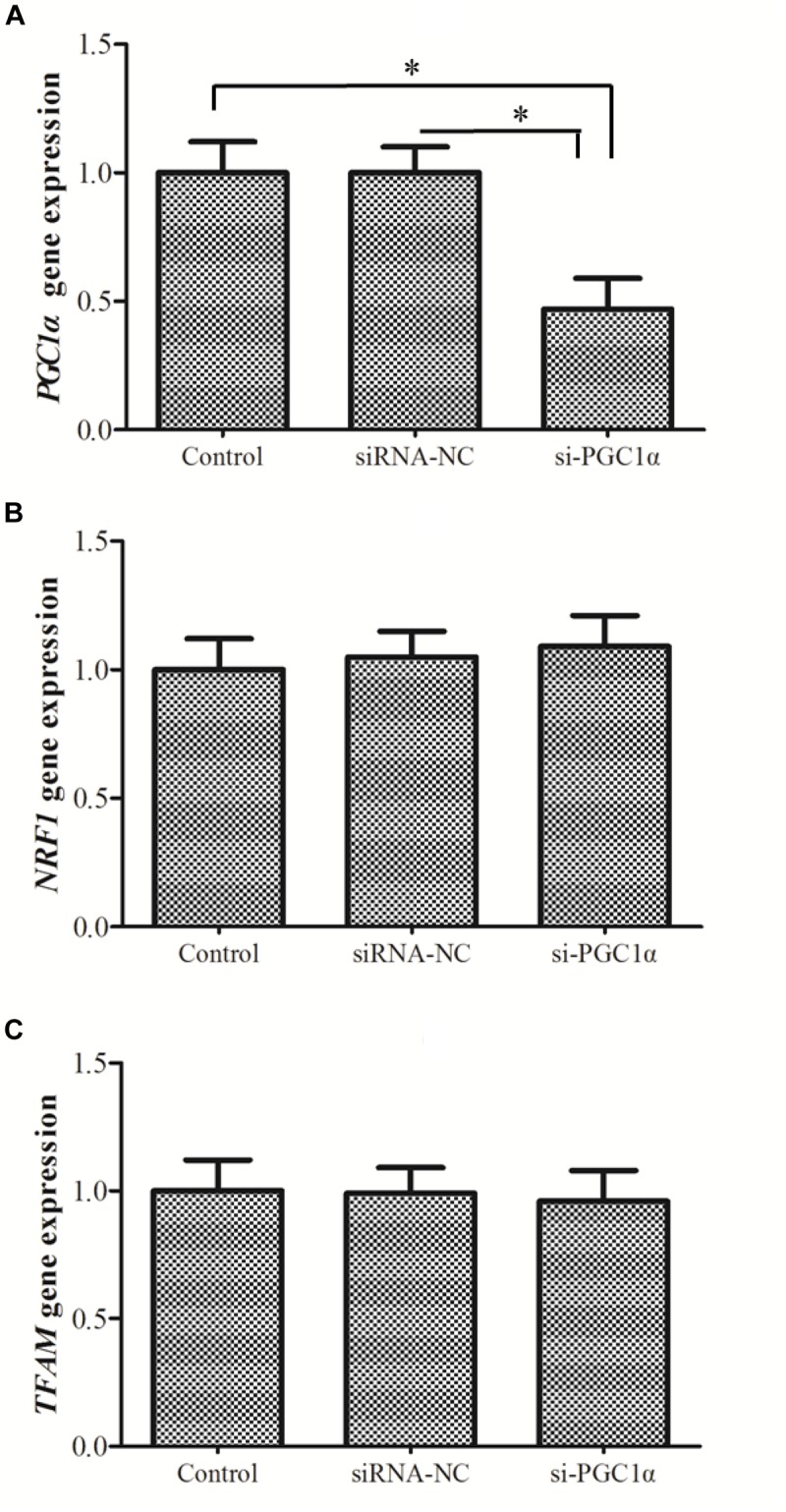
The expression of *PGC1*α **(A)**, *NRF1* **(B)**, and *TFAM* **(C)** after the knockdown of *PGC1*α. siRNA-NC, negative control; si-PGC1α, siRNA-PGC1α group. One-way analysis of variance (ANOVA) with *post hoc* multiple comparison by Student–Newman–Keuls test was used to analyze differences. The asterisk indicate significant difference (*P* < 0.05).

Knockdown of *PGC1*α did not alter *NRF1* or *TFAM* expression (Figure 6), supporting the possibility that PGC1α does not regulate the expression of NRF1 and TFAM in hepatocytes of *M. amblycephala* (Figure 6).

### Mitochondrial Biogenesis in Oleic Acid Treated Hepatocytes

**FIGURE 7:**
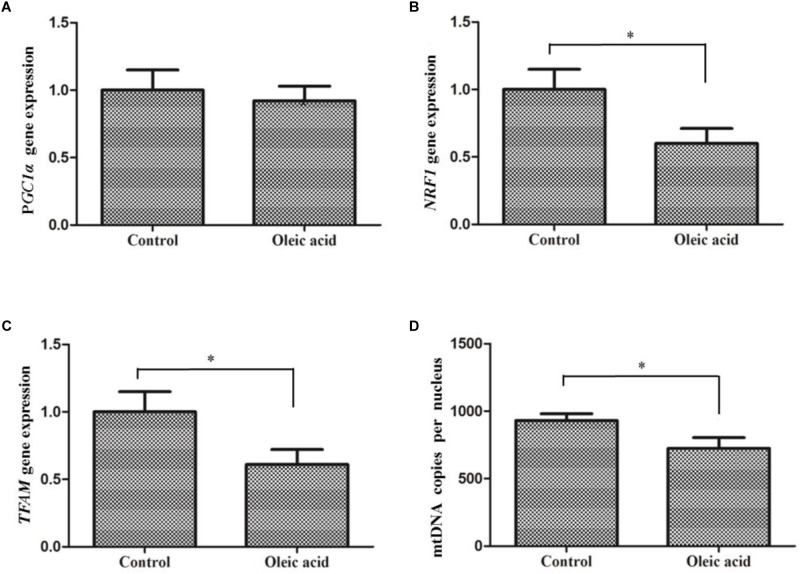
The expression of *PGC1*α **(A)**, *NRF1* **(B)**, and *TFAM* **(C)** and mtDNA copy number **(D)** in primary hepatocytes of *M. amblycephala* treated with oleic acid. Student’s *t*-test was used to analyze differences and the asterisk indicate significant difference with Control group (*P* < 0.05).

Treatment of hepatocytes with oleic acid significantly down-regulated the expression of *NRF1* (*P* = 0.002) and *TFAM* (*P* = 0.001) compared to the control group (Figure 7). Moreover, mtDNA copy number in the oleic acid treated group was significantly lower than (*P* = 0.01) that of the control group. But, *PGC1*α expression did not significantly differ between oleic acid and control groups.

## Discussion

In mammals, PGC1α acts as a positive regulator of mitochondrial biogenesis and homeostasis, adaptive thermogenesis, gluconeogenesis, and some other metabolic processes (Handschin and Spiegelman, 2006). Although orthologs of PGC1 family members have been found in fish species, its molecular characterization and functional analysis have been less studied (Lin et al., 2005). The ratio of non-synonymous/synonymous substitution rates (ω = dn/ds) detected by Codeml software of PAML package can provide a measure for the change of selection pressures. Values of ω = 1, <1, and >1 indicate neutral evolution, purifying selection, and positive selection on the target gene, respectively. Positive selection plays an important role in gene duplication, functional diversification and genomic structural divergence (Misha and Dan, 2010; Song et al., 2012). Interestingly, we found a branch underwent positive selection when the fish evolved to amphibians, birds and mammals (ω = 1.27) suggesting that the function of PGC1α might differ between fish and other vertebrates. Moreover, the positive selection site also means mutation site which could affect the function of genes (Misha and Dan, 2010). The sequence in the human homologue containing an AMPK-phosphylated Thr (RIRTNP) differs from that of the PGC1α sequence identified from blunt snout bream (RIRPTP). Further, in some fish species, the Thr is not present in this sequence. Our results support the conjecture that the function divergence of PGC1α between fish and other vertebrates might be due to the positive selection in evolution of PGC1α.

Fish muscle contains great quantities of mitochondria, myoglobin and glycogen, and plays important roles in carbohydrate and lipid metabolism (Greek-Walker and Pull, 2010). Due to the crucial role of PGC1α in metabolism its expression level in muscle is often high. Meanwhile, expression of *PGC1*α in fat tissue was higher than liver (the main tissue for fatty acid oxidation in fish) which supports the role of PGC1α in differentiation and development of adipocytes (Waldman et al., 2016).

PGC1α is involved in regulating mitochondrial content in mammals and birds (Handschin and Spiegelman, 2006). In the current study, mtDNA copy number in different tissues revealed no correlation between *PGC1*α expression and mtDNA copy number. Furthermore, the results obtained by siRNA technology indicated that knockdown of *PGC1*α does not result in reduction of mtDNA copy number in primary hepatocytes of *M. amblycephala*. This is in contrast with the results of studies on mammalian cells where down-regulation of *PGC1*α often resulted in a robust decrease of mitochondrial number (Janice et al., 2002). These observations indicate that PGC1α does not appear to participate in the regulation of mitochondrial biogenesis in *M. amblycephala*, which is in agreement with previous findings (LeMoine et al., 2008; Bremer et al., 2012). This finding suggests a functional divergence between fish and mammals. In the context of metabolic remodeling in fish muscle in response to exercise and stressors, mitochondrial changes occurred in a PGC1α independent manner, possibly compensated through changes in PGC1β (McClelland et al., 2006; LeMoine et al., 2008). As the PGC1 family has three members (PGC1α, PGC1β, PRC), it is possible that another member may participate in the regulation of mitochondrial biogenesis in fish.

Mitochondrial metabolism provides the majority of the energy within eukaryotic cells, and AMPK-PGC1α-NRF1 pathway is thought to play a key role in this process (Scarpulla, 2011; Austin and St-Pierre, 2012; Picca and Lezza, 2015). In mammals, AMPK is an essential energy sensor and its activation is partially mediated by phosphorylating PGC1α (Narkar et al., 2008; Burkewitz et al., 2014). Also, fish AMPK is activated after diet intake and exercise (Fuentes et al., 2013; Magnoni et al., 2014). Mammalian AMPK phosphorylates PGC1α at Thr177 and Ser538 sites and activates *PGC1*α expression (Jäger et al., 2007). Phosphorylation of PGC1α by AMPK promotes mitochondrial biogenesis in murine muscle cells, and the mutation of both regulatory sites ablates PGC1α coactivating activity on its own promoter (Jäger et al., 2007). Moreover, it has been reported that there is an auto-regulatory loop whereby more active PGC1α induces its own transcription (Handschin et al., 2003). Thus, phosphorylation of PGC1α by AMPK often enhances *PGC1*α expression (Frier et al., 2012). In the present study, expression of *PGC1*α was assessed after AMPK activation/inhibition in hepatocytes and the result showed no significant alteration in *PGC1*α expression. This is consistent with previous reports indicating that there is no correlation between AMPK activity and *PGC1*α expression in fish (Bremer et al., 2016). PGC1α of *M. amblycephala* lacks the critical Thr177, which could be the reason for the absence of correlation between AMPK activation/inhibition and *PGC1*α expression.

PGC1α exerts its effects on the muscle phenotype via NRF1, NRF2 and myocyte enhancing factor 2c (MEF2c). It binds these transcription factors through independent motifs and mediates their respective roles in muscle metabolism (Wu et al., 1999; Michael et al., 2001; Lin et al., 2002; Vercauteren et al., 2008). Considered together, these features garnered PGC1α the label of a control gauge of oxidative metabolism in mammals (Puigserver and Spiegelman, 2003; Scarpulla, 2006). PGC1α activates the transcription of *NRF1* which binds specifically to *TFAM* promoter (a direct regulator of mtDNA replication) (Austin and St-Pierre, 2012; Picca and Lezza, 2015). NRF1 can bind to numerous gene promoters to regulate mitochondrial functions such as mtDNA transcription and replication (Choi et al., 2002; Signorile et al., 2014). In general, NRF1 and PGC1α can bind together to form a heterodimer by an ill-defined region spanning amino acids 180–403 (Scarpulla, 2002). The binding of NRF1 and PGC1α can enhance the transcription of NRF1. Thus, in mammals, PGC1α can profoundly induce alterations in *NRF1* and *TFAM* expression (Wu et al., 1999). However, our present results showed fish *PGC1*α did not affect *NRF1* and *TFAM* expression. This can be explained by that fish PGC1α has a Ser- and Gln-rich insertions of 12–31 residues in the interaction domain and cannot bind with NRF1 (Bremer et al., 2016).

Mitochondrial biogenesis is a noticeable response of hepatocytes to a variety of physiological changes (Hood et al., 2000). Several dietary components such as resveratrol (Csiszar et al., 2009) and fatty acids (García-Ruiz et al., 2015; Lee et al., 2016) influence the mitochondrial biogenesis. It has been shown that EPA and DHA can modulate mitochondrial biogenesis which is linked to increased mtDNA replication and *PGC1*α expression (Lee et al., 2016). In this study, mtDNA copy number significantly decreased in oleic acid treated group. This was in agreement with the results of studies on mammals where saturated fatty acids reduced mitochondrial mass and caused mitochondrial dysfunction (García-Ruiz et al., 2015). Moreover, results of previous *in vivo* studies showed that administration of high-fat diets reduces mitochondrial mass and activity of the related enzymes in fish (Du et al., 2010; Lu et al., 2014). In order to clarify the underlying mechanism of mitochondrial biogenesis, expression of the genes involved in mitochondrial biogenesis in hepatocytes such as *PGC1*α, *NRF1*, and *TFAM* were examined in this study. Decreased expression of *NRF1* and *TFAM* by oleic acid treatment indicated that oleic acid regulates mitochondrial mass potentially through mtDNA replication. However, *PGC1*α expression was not significantly influenced by oleic acid, and confirmed the notion that there is no correlation between *PGC1*α expression and mitochondrial content in hepatocytes of blunt snout bream.

## Conclusion

The data generated in this study indicated that *PGC1*α does not take part in mitochondrial biogenesis in hepatocytes of blunt snout bream.

## Author Contributions

KL and XS conceived and designed the experiments. KL, WZ, and LC performed the experiments. KL, SR, and XS analyzed the data and wrote the manuscript. KL, LC, and WZ contributed reagents, materials, and analysis tools.

## Conflict of Interest Statement

The authors declare that the research was conducted in the absence of any commercial or financial relationships that could be construed as a potential conflict of interest.

## Footnotes

1 http://smart.embl-heidelberg.de/
