# Supplementary material for: Molecular Characterization of Peroxisome Proliferator-Activated Receptor-Gamma Coactivator-1α (PGC1α) and Its Role in Mitochondrial Biogenesis in Blunt Snout Bream (Megalobrama amblycephala)
Source: Front Physiol. 2019 Jan 24;9:1957. doi: 10.3389/fphys.2018.01957 (PMC6354234; doi:10.3389/fphys.2018.01957)
Supplement: Supplementary file 1 [file Table_1.DOCX]

Supplementary Material

**Molecular characterization of** **Peroxisome Proliferator-activated Receptor Coactivator-1α (PGC-1α) and** **its role in mitochondrial biogenesis in** **blunt snout bream** ***Megalobrama amblycephala***

Xiaojun Song^1†^, Samad Rahimnejad^2,3†^, Wenhao Zhou^2^, Linsen Cai^2^, Kangle Lu^2,^*

^1^ Laboratory for molecular biology of animal nutrition and immunology, College of Life Sciences, Qingdao Agricultural University, Qingdao, 266109, China

^2^ Laboratory of Aquatic animal nutrition and physiology, Fisheries College, Jimei University, Xiamen 361021, China

^3^ University of South Bohemia in Ceske Budejovice, Faculty of Fisheries and Protection of Waters, South Bohemian Research Center of Aquaculture and Biodiversity of Hydrocenoses, Institute of Aquaculture and Protection of Waters, Na Sádkách 1780, 370 05 České Budějovice, Czech Republic

^†^These authors contributed equally to this work. *Corresponding Author: lukangle@jmu.edu.cn

Table S1 The primers used for cloning and expression analysis.

| Primers | Sequence（5’—3’） | Use |
| --- | --- | --- |
| Primers used for RACE | | |
| PGC1α5-R | CTCAGAGAGGTCAAGC | Synthesis of the first-strand cDNA for 5’RACE |
| PGC1α5-R1 | AGGCAGGTCAGGGCAAAG | Used with Oligo(dT)16AP for first PCR of 5’RACE |
| PGC1α5-R2 | ACCAAGGCAGCACACTCT | Used with AP for nested PCR of 5’RACE |
| PGC1α3-F1 | TATGCCAACTCCTCCATCAACCCCA | Used with AP for first PCR of 3’RACE |
| PGC1α3-F2 | AAGGTGAGGGACAGCAGCGTGAC | Used with RACE3-R for nested PCR of 3’RACE |
| Primers for real time PCR Amplification Tm (°C)  Efficiency (%) | | |
| PGC1α-F | TGCCCTCGGTTCATTGTC | 96 60 |
| PGC1α-R | GATTTCTGATTGGTCGCTGTA |  |
| NRF1-F | CTCTACGCCTTTGAGGACCAG | 97 60 |
| NRF1-R | CCAGTGCCAACCTGTATGAGC |  |
| TFAM-F | CTTTGGTATCCAGGGAGCAGT | 99 60 |
| TFAM-R | GTTGAATCGCATCCAGTCGT |  |
| Rpl13a -F | TCTGGAGGACTGTAAGAGGTATGC | 101 60 |
| Rpl13a -R | AGACGCACAATCTTGAGAGCAG |  |

Rpl13a : reference gene
